# AVP deficiency (central diabetes insipidus) following immunization with anti-COVID-19 BNT162b2 Comirnaty vaccine in adolescents: A case report

**DOI:** 10.3389/fendo.2023.1166953

**Published:** 2023-04-18

**Authors:** Cristina Partenope, Quincy Pedranzini, Antonella Petri, Ivana Rabbone, Flavia Prodam, Simonetta Bellone

**Affiliations:** ^1^ Division of Pediatrics, Department of Health Sciences, University of Piemonte Orientale, Novara, Italy; ^2^ Endocrinology, Department of Translational Medicine, University of Piemonte Orientale, Novara, Italy

**Keywords:** AVP deficiency, diabetes insipidus, COVID-19, SARS-CoV-2 vaccination, vaccine, pituitary stalk thickening, BNT162b2 Comirnaty vaccine, adolescent

## Abstract

**Introduction:**

The coronavirus disease 19 (COVID-19) pandemic has prompted the development of new vaccines to reduce the morbidity and mortality associated with this disease. Recognition and report of potential adverse effects of these novel vaccines (especially the urgent and life-threatening ones) is therefore essential.

**Case presentation:**

A 16-year-old boy presented to the Paediatric Emergency Department with polyuria, polydipsia and weight loss over the last four months. His past medical history was unremarkable. Onset of symptoms was referred to be few days after first dose of anti-COVID-19 BNT162b2 Comirnaty vaccine and then worsened after the second dose. The physical exam was normal, without neurological abnormalities. Auxological parameters were within normal limits. Daily fluid balance monitoring confirmed polyuria and polydipsia. Biochemistry laboratory analysis and urine culture were normal. Serum osmolality was 297 mOsm/Kg H_2_O (285-305), whereas urine osmolality was 80 mOsm/Kg H_2_O (100-1100), suggesting diabetes insipidus. Anterior pituitary function was preserved. Since parents refused to give consent to water deprivation test, treatment with Desmopressin was administered and confirmed ex juvantibus diagnosis of AVP deficiency (or central diabetes insipidus). Brain MRI revealed pituitary stalk thickening (4 mm) with contrast enhancement, and loss of posterior pituitary bright spot on T1 weighted imaging. Those signs were consistent with neuroinfundibulohypophysitis. Immunoglobulin levels were normal. Low doses of oral Desmopressin were sufficient to control patient’s symptoms, normalizing serum and urinary osmolality values and daily fluid balance at discharge. Brain MRI after 2 months showed stable thicken pituitary stalk and still undetectable posterior pituitary. Due to persistence of polyuria and polydipsia, therapy with Desmopressin was adjusted by increasing dosage and number of daily administrations. Clinical and neuroradiological follow-up is still ongoing.

**Conclusion:**

Hypophysitis is a rare disorder characterized by lymphocytic, granulomatous, plasmacytic, or xanthomatous infiltration of the pituitary gland and stalk. Common manifestations are headache, hypopituitarism, and diabetes insipidus. To date, only time correlation between SARS-CoV-2 infection and development of hypophysitis and subsequent hypopituitarism has been reported. Further studies will be needed to deepen a possible causal link between anti-COVID-19 vaccine and AVP deficiency.

## Introduction

At the end of 2019 severe acute respiratory syndrome coronavirus 2 (SARS-Cov-2) infection, named COVID-19, outbroke and plagued our healthcare systems, causing more than 6 million deaths worldwide over the last three years (1). We have learned that COVID-19 is a primarily respiratory disease, however it can affect nearly every organ system, including endocrine system (2, 3). Here, the expression of angiotensin-converting enzyme 2 (ACE2) receptors and transmembrane protease serine 2 (TMPRSS2) on many endocrine cells seems to play a crucial role in the direct pathogenetic mechanism by which the virus infects these organs (4).

Since no specific treatment was available, the pandemic urged scientists to develop targeted vaccines to face the high mortality rate of the disease. SARS‐CoV‐2 vaccines are generally safe and effective in preventing COVID‐19 severe symptoms. Injection site reactions, fever, headache, myalgia, and skin rash are the most common vaccine side effects (5). Surprisingly, some cases of endocrinopathies have occurred even after anti-COVID-19 vaccines in adults (6–8), suggesting cytokine release syndrome exacerbated by the vaccine as the possible underlying mechanism for the disease, as a result of endocrine cells susceptibility to elevation of pro-inflammatory molecules (9). To the best of our knowledge, only a few cases of AVP deficiency, also known as central diabetes insipidus (CDI) occurring after anti-COVID-19 vaccines have been cited in literature until now (10–12) and still no one in children. Here we report the first case of new onset CDI in a pediatric patient after BNT162b2 mRNA COVID-19 vaccine.

## Case presentation

**Figure 1:**
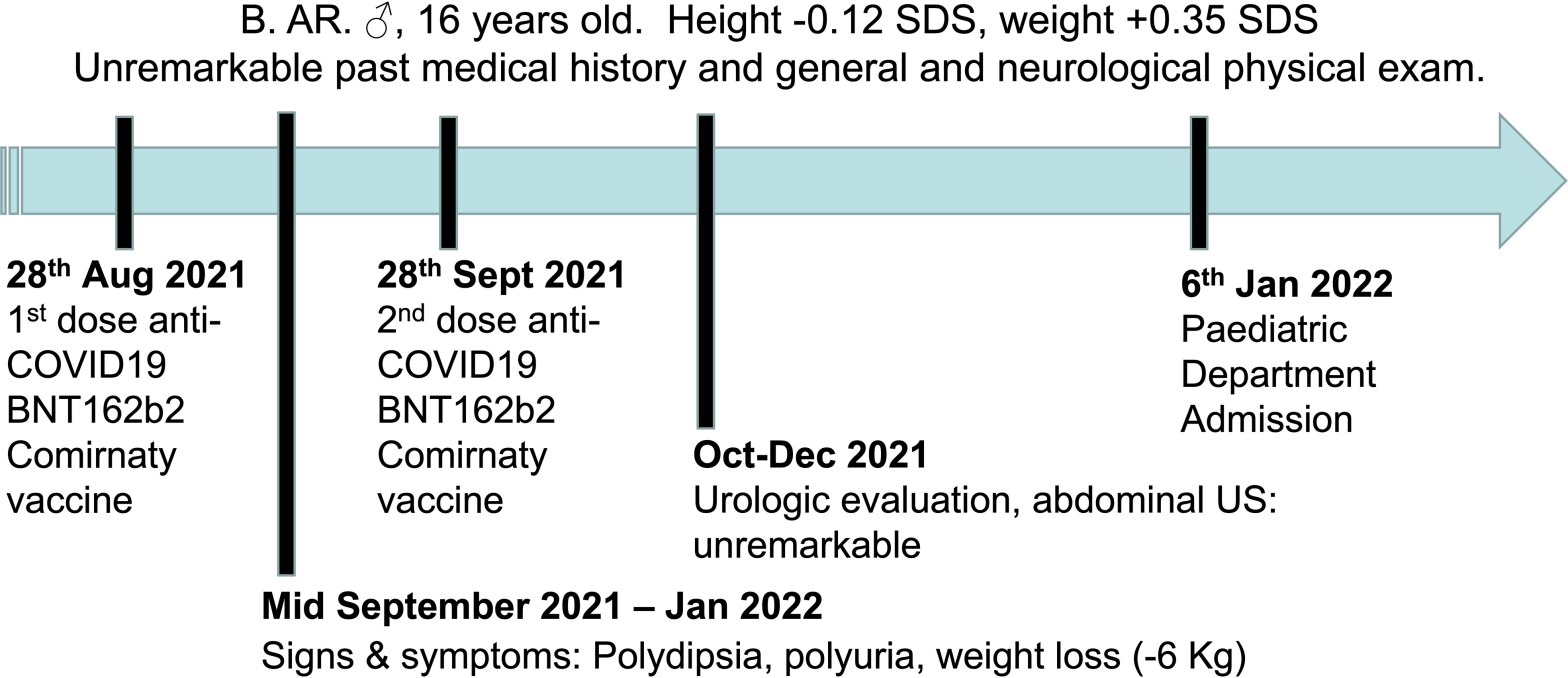
Timeline depicting the clinical course of the disease, from the onset of signs and symptoms until admission to the Paediatric Department.

B.AR., a 16-year-old boy, presented to the Paediatric Emergency Department with polyuria, polydipsia and concomitant weight loss, reporting a urine output of 9 liters in the last 24 hours and a weight loss of nearly 6 kilograms in the previous four months. Symptoms seemed to start a few days after inoculation of the first dose of BNT162b2 mRNA COVID-19 vaccine (at the end of August 2021) and worsened after the second dose, which had been administered 28 days later. Due to the persistence of intense thirst and polyuria the patient had already undergone some medical investigations: no abnormalities were found at urologic assessment and renal ultrasound (**Figure 1**). Moreover, no significant features emerged from his past medical history, and familial history was unremarkable too. On admission to the Paediatric Emergency Department, the patient’s vital parameters included heart rate 110 bpm, temperature 36°C, oxygen saturation 99% on room air. Physical examination revealed a well-being adolescent boy, with auxological parameters within normal limits (height -0.12 standard deviations, weight +0.35 standard deviations), cardiac and pulmonary auscultation without pathological findings and no neurological abnormalities (including intact sense of smell and taste and cognition). Again, head and neck/gastrointestinal/musculoskeletal examinations were all grossly unremarkable. Furthermore, biochemistry laboratory analysis, venous blood gas analysis, and urine analysis were performed (**Table 1**) and no significant alterations were found. Diabetes mellitus was excluded by detection of normal hematic glucose levels and by the absence of glycosuria. In contrast, diabetes insipidus could be suspected because of plasma sodium level at the upper limit of normal and low urine specific gravity. Given this hypothesis, the patient was tested for SARS-CoV-2 ongoing infection by nasopharyngeal swab – which resulted negative – and was then admitted to the pediatric ward to confirm the diagnosis.

**Table 1:** Results from blood and urine tests.

Lab test	Patient result	Normal ranges
SERUM CREATININE	0.69 mg/dL	0.9-1.3
BLOOD PH	7.35	7.35-7.45
BICARBONATES	31.8 mmol/L	22-26
SERUM GLUCOSE	88 mg/dl	70-100
PLASMA CALCIUM	9.36 mg/dL	8.5-10.5
PLASMA SODIUM	145 mmol/L	135-145
PLASMA POTASSIUM	4.5 mmol/L	3.5-5
C-REACTIVE PROTEIN	1 mg/dL	0-1
URINE PH	6	5.5-6.5
URINE SPECIFIC GRAVITY	1002	1005-1020
URINE HAEMOGLOBIN, GLUCOSE, PROTEIN	absent	
TSH	1.308 uIU/mL	0.450 - 3.500
FT4	1.35 ng/dL	0.89 -1.76
FT3	3.20 pg/mL	2.30 - 4.20
ACTH	28.2 pg/mL	3.6 - 60
CORTISOL	9 microg/dL	4.5 - 24
PROLACTIN	41.3 mIU/L	44.5 - 375.0
LH	2.50 mIU/mL	1.5 - 34.6
FSH	2.6 mIU/L	1.4 - 18.1
TESTOSTERONE	539.9 ng/dL	144.0 - 842.0
AFP	4.1 IU/mL	0.0 - 7.0
HCG	<2 mIU/mL	0 - 5

TSH, thyroid stimulating hormone; fT4, thyroxine; fT3, free triiodothyronine; ACTH, adrenocorticotropic hormone; LH, luteinizing hormone; FSH, follicle stimulating hormone; AFP, alpha-fetoprotein; hCG, human chorionic gonadotropin.

## Management and outcome

**Figure 2:**
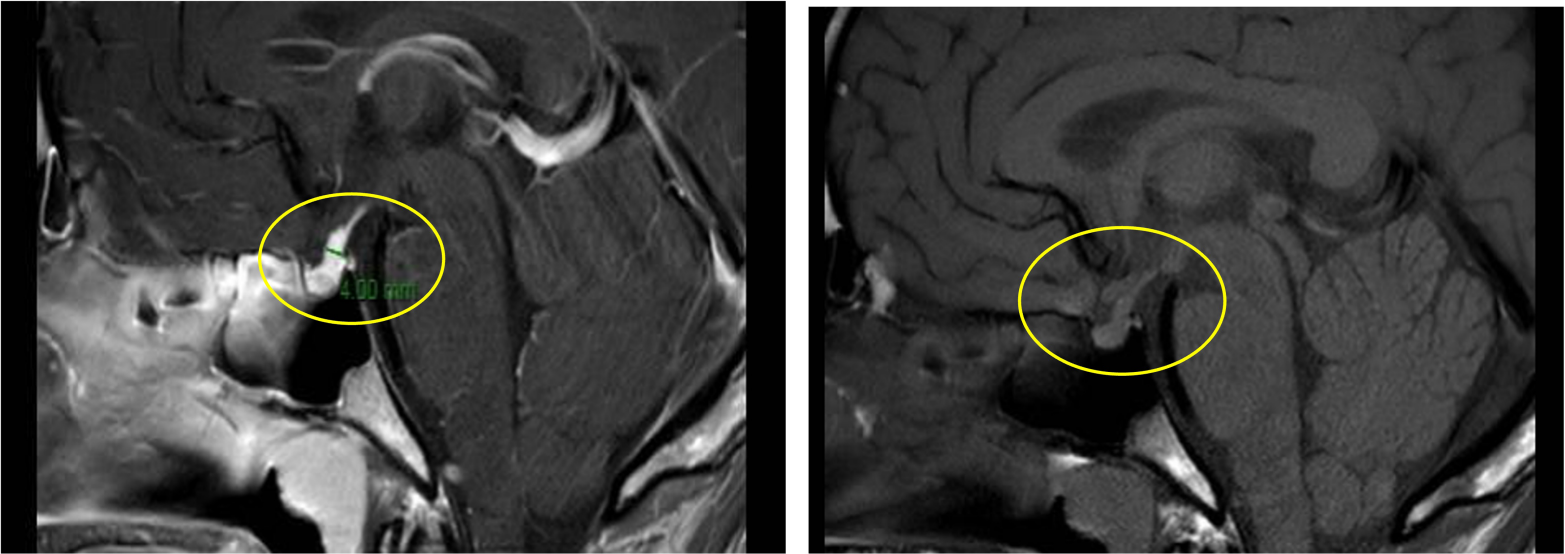
Pituitary stalk enlargement with contrast enhancement (left panel, yellow circle); loss of posterior pituitary bright (right panel, yellow circle).

**Figure 3:**
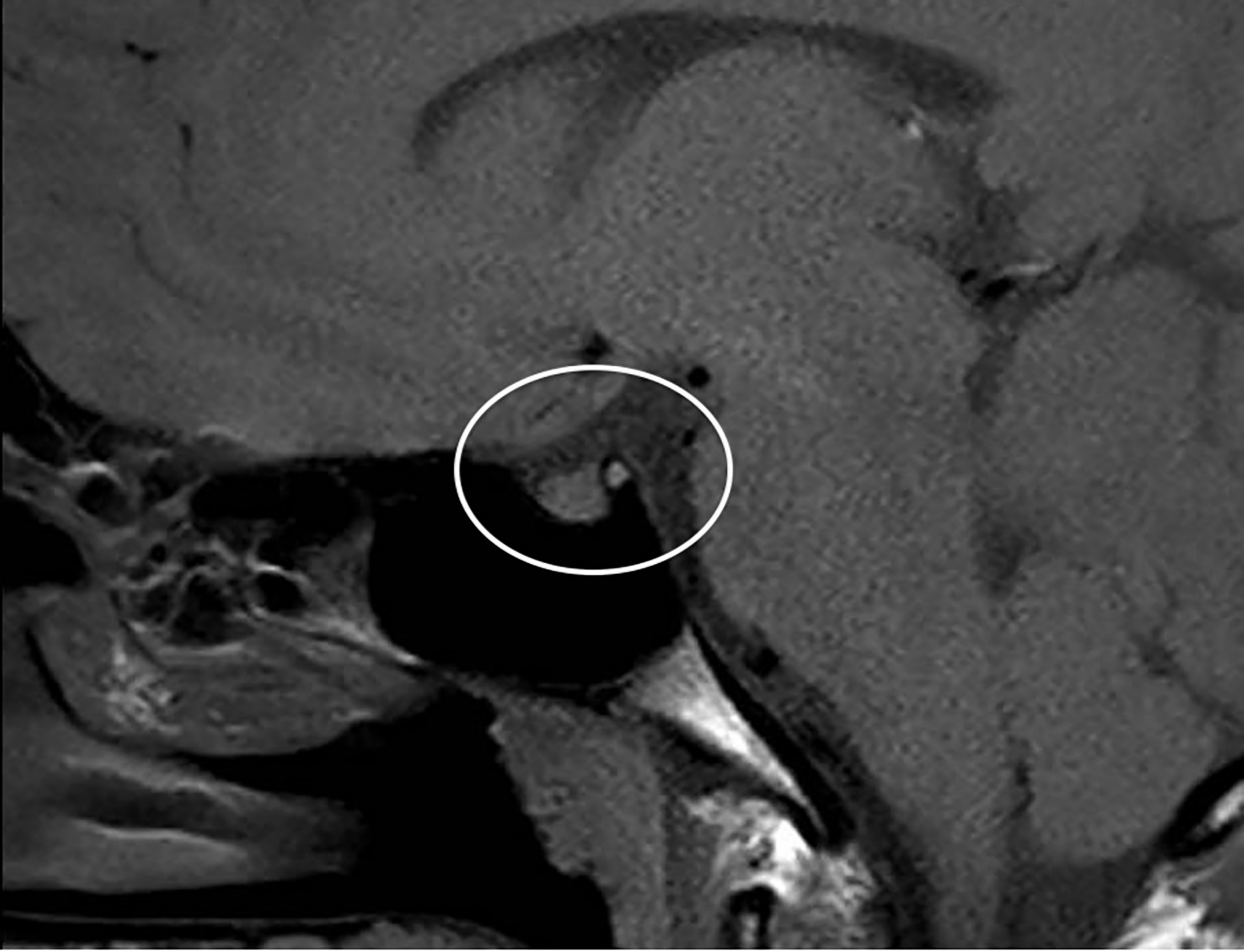
Brain MRI after 2 months: stable thickened pituitary stalk and undetectable posterior pituitary (circle).

Primarily, daily fluid balance monitoring confirmed polyuria and polydipsia (IN 6.250 L/OUT 7.100 L). Secondly, serum osmolality (p-Osm) levels were 287 mOsm/Kg H_2_O (normal values 285-305), urine osmolality (u-Osm) was 68 mOsm/Kg H_2_O (normal values 100-1100) and u-Osm/p-Osm ratio was < 1, so that diabetes insipidus could be suspected. However, parents did not give consent to water deprivation test. A test with desmopressin (the synthetic analog of antidiuretic hormone) was then performed to discern between central and nephrogenic DI. After administration of low dose oral desmopressin (sublingual 60 micrograms) an immediate response was evident in view of both normalization of daily fluid balance (IN 0.520 L/OUT 0.600 L) and rapid increase of u-Osm (119 mOsm/Kg H_2_O). Consistent with those findings, ex juvantibus diagnosis of complete CDI could be made. To complete investigations, hormonal tests showed no significant impairment of anterior pituitary function (**Table 1**), immunological assessment revealed normal immunoglobulin levels (IgA, IgM, and IgG subclasses, including IgG4) and urine culture and Quantiferon were negative as well. Brain contrast-enhanced magnetic resonance imaging (MRI) focused on the study of the pituitary region was carried out. It revealed pituitary stalk thickening (PST) (maximum diameter of 4 mm) with contrast enhancement and loss of posterior pituitary bright spot on T1 weighted imaging (**Figure 2**). Those signs were consistent with neuroinfundibulohypophysitis. In the view of strict time correlation between COVID-19 vaccine and onset of symptoms and granted that no other differential diagnosis had fit the clinical picture, the case was signaled as an adverse drug reaction to the Italian Pharmacological Agency (AIFA). During hospitalization, B.AR. maintained good general conditions. He was discharged after 7 days with prescription of low doses of oral desmopressin (60 mcg twice daily), as the same dose had been sufficient to control signs and symptoms during hospitalization. After 2 months, follow-up MRI showed stable PST and still undetectable posterior pituitary, consistent with the persistence of inflammation of this cerebral area (**Figure 3**). An endocrinological follow-up was also planned at our center: three months after discharge, due to persisting polyuria and polydipsia, therapy with desmopressin was adjusted by increasing dosage and number of daily administrations (60 mcg three times a day). Clinical and neuroradiological follow-up is still ongoing.

## Discussion

It is now widely accepted that COVID-19 is a multiorgan disease, as many tissues and organs are affected during ongoing or recent infection by SARS-CoV-2 virus, including endocrine system (13). It is becoming well established that in some cases SARS-CoV-2 infection can trigger an inflammation of the hypothalamus-pituitary axis (hypophysitis) both in adults and in children, resulting in a dysfunction that causes diabetes insipidus, either alone (14–17) or associated with anterior hypopituitarism (2, 14) with a latency of 0–16 weeks between recognition of the SARS-CoV-2 infection and development of symptoms (18). Although the exact pathogenetic mechanism has still to be defined, a direct pathway (involving ACE2-mediated hypothalamic viral infection) as well as an indirect, delayed, and immune-mediated pathway have been hypothesized. Moreover, endothelial damage in the blood–neuron interface, thrombotic microangiopathy (pituitary apoplexy), infected leukocyte‐mediated transportation and cytokine storming are other accepted theories (19).

Interestingly, hypopituitarism might have a bidirectional relationship with COVID-19 since pre-existent hypopituitarism can be per se a potential risk factor for COVID-19 due to its comorbidities (e.g. hypothalamic obesity) and can be worsened by SARS-CoV-2 infection (20). Moreover, COVID-19 vaccine can affect endocrine system, albeit infrequently and with good prognosis. Pezzaioli et al. recently collected and reviewed all published data on potential endocrine adverse effects post-COVID-19 vaccines in adult patients (6). Thyroid disorders are the most common; only eight cases of pituitary dysfunction have been described so far (10–12, 21–25), of which two presented with CDI (11, 12).

Our patient complained of exacerbation of DI symptoms after the second dose of COVID-19 vaccine. Although we were not able to conduct dose-specific analyses, previously published cases also reported onset or worsening of adverse endocrinological events following the second dose of vaccine (5, 22, 23). Mechanisms of increased reactogenicity after the second dose are largely unknown. One possibility is that the vaccine directly might cause pituitary impairment with cumulative effect. Autoimmune/inflammatory syndrome induced by vaccine adjuvants (ASIA syndrome), molecular mimicry, cross-reactivity or a pro-inflammatory state induced by vaccine components and subsequent activation of autoreactive B and T cells are speculated to be involved, as in Guillain-Barrè syndrome or optic neuritis (26–28).

COVID-19 symptoms are generally milder in the pediatric age than in the adult population, but this is not always true when the endocrine system is involved. Lizzi et al. described a pediatric case of acute onset of isolated CDI associated with recent SARS-CoV-2 infection (16), requiring 7 days of hospitalization. Here we described the case of an adolescent who developed CDI after COVID-19 vaccination, in whom posterior pituitary function has not recovered yet. Diabetes insipidus is a tricky disease whose symptoms can be underestimated by both patients and clinicians. CDI is characterized by decreased or absent secretion of antidiuretic hormone (ADH; also called arginine vasopressin or AVP), resulting in a variable degree of polyuria. Lack of AVP can be caused by disorders or lesions in the hypothalamic osmoreceptor, in the supraoptic or paraventricular nuclei, in the superior portion of the supraopticohypophyseal tract or in the pituitary sella. Recently, a panel of experts from national and international endocrinology and endocrine pediatric societies has proposed to change diabetes insipidus’ name to “arginine vasopressin deficiency (AVP-D)” for central etiologies to avoid detrimental confusion with diabetes mellitus for both patients and their caretakers (29).

Finally, pituitary stalk is a funnel-shaped structure that connects the hypothalamus to the pituitary gland. There are several etiologies that give rise to PST (30, 31), which often manifests clinically with CDI (32, 33): autoimmunity/inflammation (neuroinfundibulohypophysitis (34)) – sometimes referred as idiopathic PST, infectious diseases (e.g. tuberculosis (35)) or neoplastic lesions (33, 36). Anti-pituitary, anti-hypothalamus autoantibodies or high IgG4 levels have been detected in some patients with hypophysitis. However, their causal role remains unclear (37). Searching for these antibodies may help to diagnose an autoimmune hypophysitis, especially in cases like ours presenting with non-diagnostic pituitary MRI or hypoprolactinemia (38, 39). Unfortunately, no autoantibodies testing was performed in this case report since the lab kit was not available in our institution. The approach to the neuroradiological finding of PST is still controversial (32, 40): empirical management recommend to conduct follow-up MRI every 3-6 months and proceed with pituitary stalk biopsy only in case of stalk size ≥ 7 mm, progressive infundibular enlargement or worsening of symptoms. Moreover, all patients with pituitary stalk lesions and CDI should be routinely assessed for anterior pituitary hormonal function, which was normal in our patient.

## Conclusion

To date, CDI following anti-COVID-19 vaccine remains a rare and only temporally linked occurrence, even though its pathophysiological explanation has been hypothesized. Further studies will be needed to deepen a possible causal link between the two events.

## Data availability statement

The raw data supporting the conclusions of this article will be made available by the authors, without undue reservation.

## Ethics statement

Written informed consent was obtained from the individual(s), and minor(s)’ legal guardian/next of kin, for the publication of any potentially identifiable images or data included in this article.

## Author contributions

CP and QP drafted the manuscript. AP, IR, and FP were involved in the clinical management of the patient and critically revised the manuscript. SB supervised the whole process. All authors contributed to the article and approved the submitted version.
